# The impact of interprofessional education on students' current and desired competence in diabetes care

**DOI:** 10.1002/nop2.1301

**Published:** 2022-07-26

**Authors:** Sanna Kangas, Tuula‐Maria Rintala, Päivi Hannula, Esa Jämsen, Ritva Kannisto, Eija Paavilainen, Pia Jaatinen

**Affiliations:** ^1^ Department of Internal Medicine Tampere University Hospital Tampere Finland; ^2^ Faculty of Medicine and Health Technology Tampere University Tampere Finland; ^3^ Tampere University of Applied Sciences Tampere Finland; ^4^ Department of Geriatrics Tampere University Hospital Tampere Finland; ^5^ School of Medicine University of Tampere Tampere Finland; ^6^ Department of Health Sciences, Faculty of Social Sciences Tampere University Tampere Finland; ^7^ The Hospital District of South Ostrobothnia Seinäjoki Finland; ^8^ Division of Internal Medicine Seinäjoki Central Hospital Seinäjoki Finland

**Keywords:** diabetes, interprofessional education, mixed methods research, undergraduate students

## Abstract

**Aim:**

To explore the impact of interprofessional education (IPE) on undergraduate nursing and medical students' knowledge, competence and targeted competence in diabetes care.

**Design:**

Mixed methods design.

**Methods:**

A voluntary IPE course of diabetes management was organized for nursing (*n* = 15) and medical (*n* = 15) students, who performed a diabetes knowledge test and self‐evaluation of diabetes competence before and after the course and were compared with non‐participating students. The participating students' focus‐group interviews were analysed using inductive content analysis.

**Results:**

The IPE course improved nursing students' diabetes knowledge and self‐evaluated competence among nursing and medical students. The baseline differences in self‐evaluated competence between the groups disappeared. The non‐participating students evaluated their competence higher than the participants, though they scored lower or equally in the knowledge test. In conclusion, IPE showed potential in increasing students' self‐evaluated competence, motivation to learn more and nursing students' diabetes knowledge, offering better prospects for future interprofessional diabetes management.

## 1 INTRODUCTION

Diabetes is a serious global health concern with nearly half a billion people living with it worldwide. This number is estimated to increase 25% by the year 2030, causing a major impact on the lives and well‐being of individuals, families and societies. (International Diabetes Federation, 2019; Saeedi et al., 2019.) Enhancing the early detection, care and self‐management of diabetes is essential in improving the individual's quality of life and reducing the global health and economic burden of diabetes (Ahola & Groop, 2013; NCD Risk Factor Collaboration, 2016). This can be accomplished by promoting an active self‐management of people with diabetes and interprofessional collaborative care provided by competent, skillful professionals (International Diabetes Federation, 2019). Managing the care of this multifaceted disease can be challenging both for the person with diabetes and for the variety of healthcare professionals involved, requiring several aspects to be taken into account simultaneously (Fredrix et al., 2018; Lee et al., 2021). The diabetes knowledge and competence of healthcare professionals and their capability in interprofessional collaboration are associated with various outcomes of diabetes care (e.g., Atsalos et al., 2019; Lee et al., 2021).

The importance of functional collaboration and teamwork of various health professionals is highlighted in the care delivery for chronic diseases and patients with complicated conditions (e.g., Körner et al., 2016). Especially, a collaborative approach is widely supported in the education and care of diabetes, to provide cost‐effective, patient‐centred and optimal care for the large group of people living with diabetes (De La Rosa et al., 2020; International Diabetes Federation, 2019; Johnson & Carragher, 2018).

## 2 BACKGROUND

Interprofessional education (IPE) is one way to enhance the knowledge and collaborative skills necessary in interprofessional healthcare settings (Reeves et al., 2017; World Health Organization, 2010). IPE is traditionally described by WHO as education where ”two or more professions learn about, from and with each other, to enable effective collaboration and improve health outcomes” (WHO, 2010, p. 13). Several positive outcomes have been attributed to IPE, such as students' improved collaborative competency (Guraya & Barr, 2018; Riskiyana et al., 2018), positive attitudes towards collaborative teamwork (Guraya & Barr, 2018) and increased knowledge of the roles of other professions (Kent & Keating, 2015; Lim & Noble‐Jones, 2018). Many institutions have highlighted the relevance of performing IPE in the healthcare context (e.g., Interprofessional Education Collaborative, 2016; WHO, 2010), as there are also notions that today's healthcare students, who are educated separately from the students of other healthcare professions, may be unprepared for the real life environments in which they will be working together after graduation (Speakman & Arenson, 2015). Positive results have been reported on IPE in diabetes management, such as improvement in students' knowledge and skills, confidence in and motivation for treating patients with diabetes and teamwork competency in diabetes management (Kangas et al., 2018). Nevertheless, there is still a need for improving interprofessional collaboration in diabetes care (Sørensen et al., 2020), in order to improve the quality of care (Stuckey et al., 2015).

To be able to educate collaboratively competent healthcare professionals for the future, it is relevant to explore students' current competence and ideas of what they desire to learn in interprofessional diabetes management and the impact of IPE in this context. Previously, IPE programmes on diabetes management have been targeted mainly to professionals (Kangas et al., 2018). Only a few studies have evaluated undergraduate students' competence or knowledge in diabetes management (Račić et al., 2017), and research on nearly graduated healthcare students' desired clinical competence is scarce (e.g., Bork, 2003). To the best of our knowledge, no previous IPE‐related studies have been published on undergraduate healthcare students' current and desired competence in interprofessional diabetes care or on their perceptions of how to achieve the desired competence.

The purpose of this study was to explore the impact of a voluntary IPE intervention on undergraduate nursing and medical students' knowledge, competence and desired competence in interprofessional diabetes care. Our research question was: What kind of knowledge, competence and perceptions of their future competence in diabetes management do nursing and medical students have, and what is the impact of diabetes‐specific IPE on these areas?

## 3 THE STUDY

### 3.1 Design

This is a mixed methods study with a convergent parallel design (Halcomb & Hickman, 2015), employing a pre‐test post‐test quantitative analysis and a qualitative approach to focus‐group interviews. The relevance of using a mixed methods design in this study is based on its ideology that combining of both qualitative and quantitative data provides deeper understanding of the research problem than a single approach (Fetters et al., 2013). The convergent parallel design was applied, i.e., the qualitative and quantitative data were collected within the same time frame, analysed and presented separately in the Results section and merged later in the interpretation phase in the Discussion section, when applicable (Halcomb & Hickman, 2015; Shorten & Smith, 2017).

### 3.2 The IPE course of diabetes management and the participants

The participants were undergraduate second‐ or third‐year nursing students (*n* = 15) and fifth‐ or sixth‐year medical students (*n* = 15), who had previously finished their mandatory studies in diabetes care. The students were enrolled in a voluntary, clinical experience and active learning oriented interprofessional course of diabetes management and an associated study (Supporting Information 1), as described in detail previously (Kangas et al., 2021). An email with information on the upcoming voluntary IPE course and the associated study was sent to the nearly graduating nursing and medical students. The first 30 voluntary participants were enrolled after a review of the entitlement for admission. The control groups included nursing students (knowledge test *n* = 27; self‐evaluation *n* = 15) and medical students (knowledge test *n* = 47; self‐evaluation *n* = 15) at the same stage of their studies, who did not participate in the course. The knowledge test was accomplished by all peer students after finishing their normal, mandatory diabetes studies, and the self‐evaluation was performed by the students who volunteered to participate in the IPE course, but were not enrolled, because the maximum number of participants had already been reached.

The course targeted at providing in‐depth knowledge of diabetes and its interprofessional care. It introduced to the students experts in different areas of diabetes care, aiming to increase the students' understanding of the roles of different disciplines in diabetes management. Diabetes specialist nurses, endocrinologists, a podiatrist, a social worker, a dietitian and a geriatrician delivered interactive lectures on subjects selected by the students and covering the main areas of interprofessional care of diabetes. In addition, the course aimed at improving the students' skills and abilities as a member of an interprofessional team. Group discussions were arranged at the beginning and at the end of the course, to share and discuss, e.g., the personal values of the students and their understanding of the roles of each profession and the patient in diabetes care.

Each nurse–physician pair of students participated in two working visits of half a day each, including clinical work in a gerontological ward and at a diabetes outpatient clinic. The students had instant feedback on their work from the experts present. Afterwards, the student pairs prepared a presentation of one of the patient cases they had met. Altogether four 2‐h interprofessional seminars were organized, including the case presentations, short presentations by the experts and discussions on the questions raised by the presentations. Self‐studies were supported by offering the students references to relevant, up‐to‐date literature on diabetes care. The extent of the course was three ETCS credits.

### 3.3 Assessment of diabetes knowledge and competence

We used a 20‐question diabetes knowledge test, based on the National Current Care Guidelines of diabetes management, before and after the course (Supporting Information 2). In addition, a web‐based tool, provided by the National Finnish Diabetes Association (Finnish Diabetes Association), was used for the self‐assessment of the students' current and targeted competence in diabetes management before and after the course. The tool includes 13 competence‐areas of interprofessional diabetes management, rated from 0 = no competence to 5 = specialized competence, as described in detail previously (Kangas et al., 2021). The control groups performed the same knowledge test and self‐evaluation once, after finishing their mandatory diabetes education. The participating students were focus‐group interviewed in three mixed groups after the course using a semi‐structured interview form. The questions of the interview were related to the purpose and goals of the study and students' perceptions of their competence in diabetes care. (Kangas et al., 2021). The question analysed in this report was: What kind of targets have you set for your future competence in diabetes management and how do you intend to achieve them?

### 3.4 Data analysis

The normality of the numerical data from the knowledge tests and the self‐evaluations was assessed visually and according to the skewness values, which in many cases indicated a non‐normal distribution (Organ, 2020). Therefore, the numerical data were presented using median, minimum and maximum values, and non‐parametric methods were used in the statistical analyses (Kühnast & Neuhäuser, 2008). In the illustrations, group means were used to visualize the overall level of self‐evaluated competence in each group, because illustrations based on the median values would have been less informative. A *p*‐value of <.05 was considered to indicate a statistically significant difference. The Wilcoxon Signed‐Rank Test was used to analyse the changes in pre‐ versus post‐course self‐evaluations and knowledge tests within the nursing and medical student groups. The comparisons between the participating vs. control groups and nursing vs. medical student groups were performed using the Mann–Whitney *U* test (SPSS software for Windows 25.0).

The focus‐group interviews were analysed using an inductive content analysis, as it presents a systematic and objective analysis of previously unknown phenomena (Elo & Kyngäs, 2008). The first author (SK) listened to the interviews and read the transcripts, and after getting an overview of the data, performed an open coding by underlining different meaning units in the text in view of the research question (Bengtsson, 2016). Repetitive meaning units of the same characteristics were shortened, coded and grouped in subcategories. Each code was compared for differences and similarities to be sorted in the adequate, descriptive category (Supporting Information 3) (Graneheim & Lundman, 2004). During the analysing process, these stages were reviewed to verify the quality and trustworthiness of the analysis and to judge the internal homogeneity and external heterogeneity of the subcategories (Bengtsson, 2016). The initial coding process of the first author (SK) was reviewed independently by another author (T‐MR) and thereafter, an agreement of the categorization was discussed to foster validity (Elo & Kyngäs, 2008). The merging of the qualitative and quantitative findings was performed by the first author (SK) and confirmed by the other authors.

### 3.5 Ethics

The course and the associated study protocol were approved by the Tampere Planning Committee of the Degree in Licentiate of Medicine. The patients involved in the students' clinic visits, and the participating and non‐participating students were informed of the study protocol, of their rights and the anonymity and confidentiality in the study. Informed consent was obtained from all the participants prior to enrollment in the study (Kangas et al., 2021). The study was conducted according to the good scientific practice guidelines (The Finnish Advisory Board on Research Integrity, 2012), and The Code of Ethics of the World Medical Association (2017). The person who analysed the data of the focus‐group interviews was not involved in the planning or implementation of the course. No formal evaluation was performed on the course, which was passed based on attendance.

## 4 RESULTS

### 4.1 Diabetes knowledge of the students

The nursing students scored overall lower than the medical students in both the pre‐course and the post‐course knowledge tests. After the course, pre‐post improvement was observed in the nursing students only. The knowledge test scores of the participating nursing students were higher than those of the non‐participating controls, but there was no significant difference in the scores of the participating vs. non‐participating medical students. In addition, the nursing students had more variation in their scores than the medical students, but the variation diminished after the course. (Table 1).

**TABLE 1:** The diabetes knowledge test scores and self‐evaluated diabetes competence of nursing (nurse) and medical (med) students before and after diabetes specific interprofessional education (IPE) course and the test scores of matched control students (control) not participating in the course

	IPE students						Control			Significance of differences (*p*)			
	Pre‐course			Post‐course			Students			Pre‐post^c^	IPE‐control^d^	Nurse‐med^d^	
	Md	Min	Max	Md	Min	Max	Md	Min	Max			Pre	Post
Diabetes knowledge test score^a^	(Nurse *n* = 14, Med *n* = 15)			(Nurse *n* = 15, Med *n* = 14)			(Nurse *n* = 27, Med *n* = 47)					.016*	.023*
Nurse	17.0	5.0	29.5	21.5	19.0	28.5	18.0	7.0	29.0	.030*	.006*		
Med	24.0	20.0	29.0	25.8	19.0	29.5	24.5	15.5	31.0	.219	.542		
Area of self‐evaluated competence^b^	(Nurse *n* = 15, Med *n* = 15)			(Nurse *n* = 14, Med *n* = 14)			(Nurse *n* = 15, Med *n* = 15)						
Diabetes prevention												.004*	.903
Nurse	2	1	3	3	1	4	4	1	4	.004*	.002*		
Med	2	2	4	3	2	4	4	3	5	.030*	<.001*		
Diabetes diagnosis & epidemiology												<.001*	.100
Nurse	2	1	2	2	1	4	4	1	4	.013*	.001*		
Med	2	2	3	3	3	4	4	3	5	.038*	.001*		
Diabetes assessment, care & follow‐up												.002*	.656
Nurse	2	0	3	3	1	4	4	1	4	.003*	.001*		
Med	2	2	3	3	3	4	4	3	5	.005*	.001*		
Medication												.001*	.066
Nurse	1	0	2	2	0	3	5	1	5	.011*	<.001*		
Med	2	1	3	3	2	4	4	3	5	.008*	.001*		
Insulin therapy												.023*	.823
Nurse	1	0	2	3	0	4	4	1	5	.010*	.001*		
Med	2	1	3	3	1	4	4	3	5	.018*	<.001*		
Nutrition												.384	1.000
Nurse	2	1	3	3	1	4	5	2	5	.006*	<.001*		
Med	2	1	3	3	1	4	4	3	5	.020*	.023*		
Exercise												.158	.526
Nurse	2	1	3	3	2	4	4	2	5	.002*	.002*		
Med	2	1	3	3	1	4	3	2	5	.034*	.072		
Foot care												.885	.229
Nurse	1	1	3	3	1	3	4	1	5	.004*	<.001*		
Med	1	1	2	2	1	4	3	2	5	.006*	.005*		
Psychology												.138	.806
Nurse	1	0	2	2.5	1	3	4	1	4	.005*	<.001*		
Med	2	0	3	2	1	4	3	2	5	.071	.007*		
Management of care & rehabilitation												.067	.447
Nurse	0	0	2	2	0	4	4	0	4	.004*	.001*		
Med	1	0	2	2	0	4	4	3	5	.010*	<.001*		
Individual patient guidance												.788	.075
Nurse	1	1	3	3	1	4	4	1	4	.003*	.009*		
Med	1.5	0	3	2	0	4	4	1	5	.031*	.002*		
Patient group guidance												.495	.405
Nurse	1	0	2	2	0	3	4	0	3	.003*	<.001*		
Med	0	0	2	2	0	4	3	0	4	.041*	.050		
Social welfare & legislation												.741	1.000
Nurse	1	0	2	2	0	3	4	1	3	.012*	.001*		
Med	1	0	2	2	1	4	3	2	5	.013*	<.001*		

Abbreviations: Md, median; Max, maximum; Min, minimum.

* Statistically significant difference, *p* < .05.

^a^ Range 0–40.

^b^ 0 = no competence, 1 = basic competence, 2 = fair competence, 3 = proficient competence, 4 = highly advanced competence, 5 = specialized competence.

^c^ Wilcoxon Signed Rank test.

^d^ Mann–Whitney *U* test.

### 4.2 Self‐evaluated current competence in interprofessional diabetes management

**FIGURE 1:**
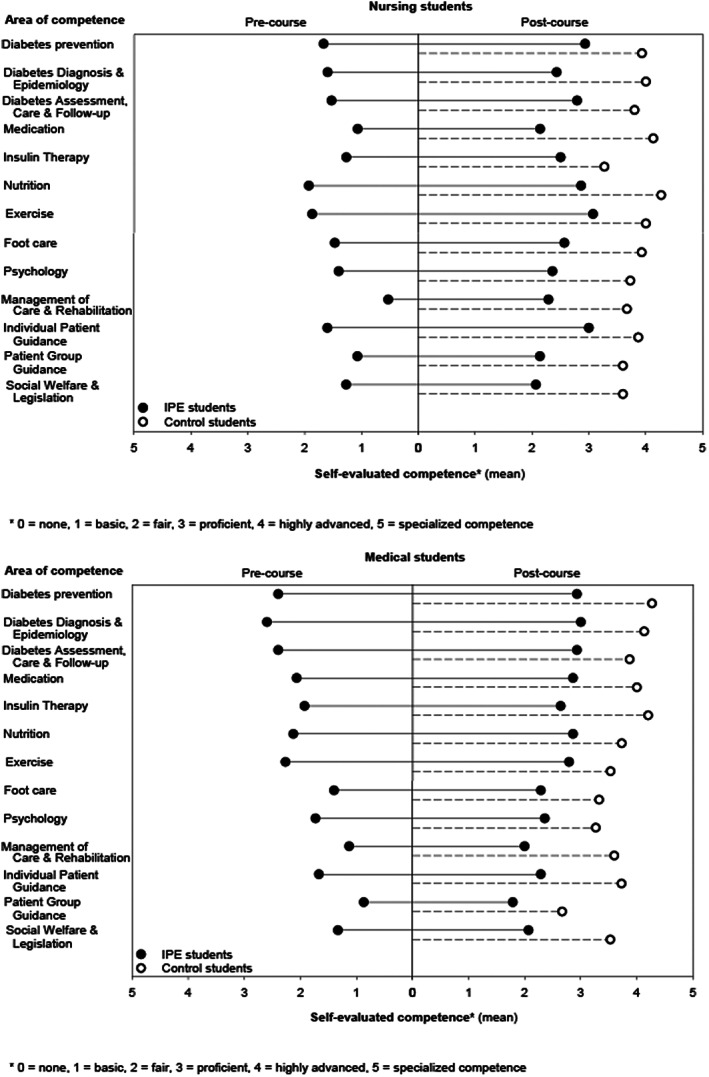
Nursing and medical students' self‐evaluated diabetes competence before and after the interprofessional education (IPE) course and compared with control students not participating in the course. Group means were used instead of medians to visualize the overall level of self‐evaluated competence in each group

Before the interprofessional course, the nursing students evaluated their competence in diabetes management lower than the medical students in five areas of competence. After the course, there were no differences between the nursing and medical students in any of the areas of self‐evaluated diabetes competence. (Table 1, Figure 1). The pre–post comparison of self‐evaluated competence indicated improvement in both groups in all areas, except for the area of Psychology in the medical students. The non‐participating control students evaluated their diabetes competence significantly higher than the participating nursing and medical students, in all except for two competence areas for the medical students (Table 1).

### 4.3 Targeted competence in interprofessional diabetes management

**FIGURE 2:**
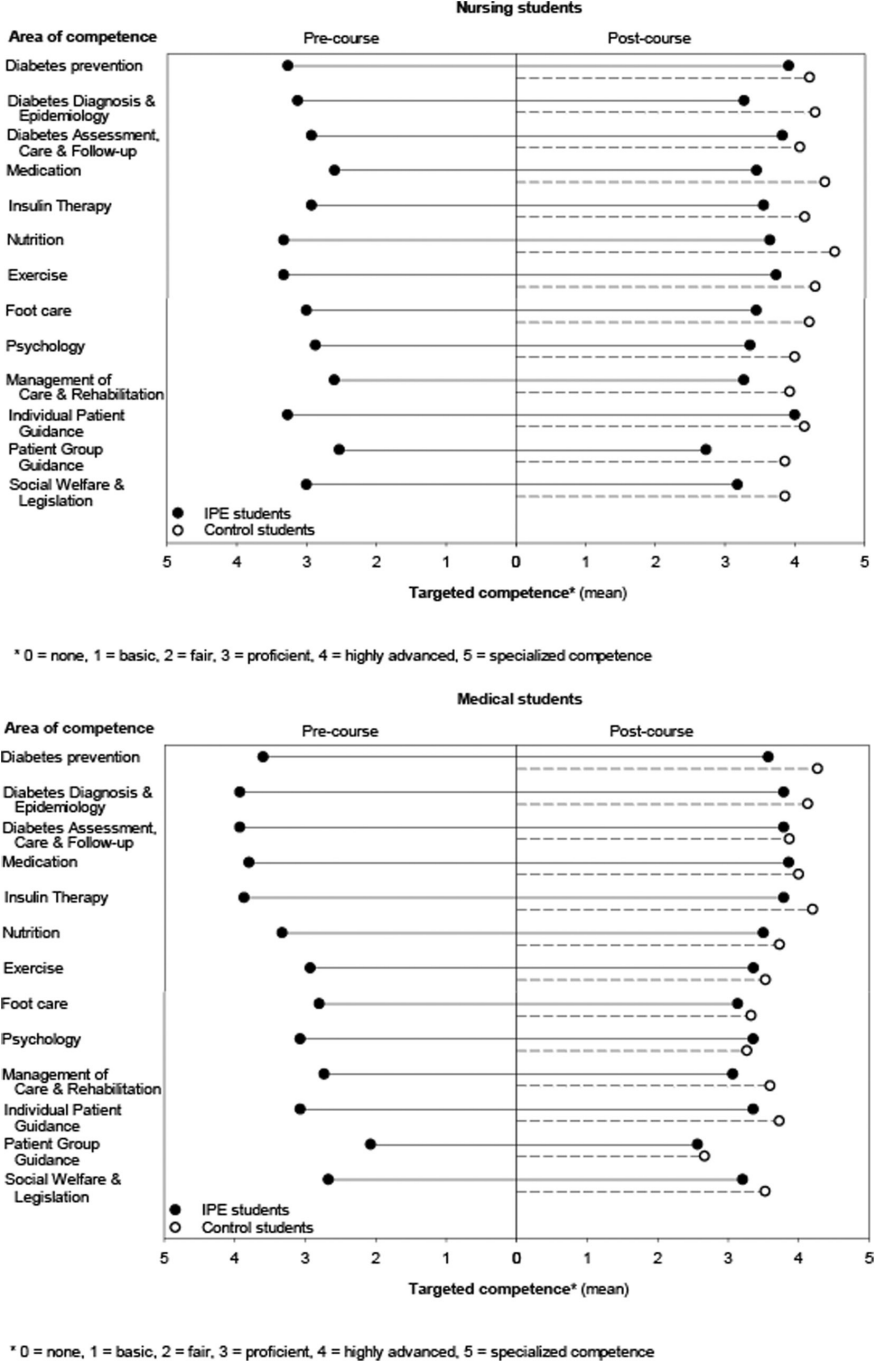
Nursing and medical students' targeted diabetes competence before and after the interprofessional education (IPE) course and compared with control students not participating in the course. Group means were used instead of medians to visualize the overall level of self‐evaluated competence in each group

After the course, there was increase in two areas of targeted competence among the medical students and in four areas of targeted competence among the nursing students, compared with the pre‐course target levels (Table 2, Figure 2). When comparing the participating nursing and medical students, the target levels were higher for medical students in four areas of competence before the course, whereas after the course, the only difference was the nursing students' higher target level in Individual Patient Guidance. Compared with the control groups, the participating nursing students set their targeted competence level lower in seven areas of competence and medical students in one area of competence (Diabetes prevention). (Table 2, Figure 2).

**TABLE 2:** The targeted diabetes competence of nursing (nurse) and medical (med) students before and after the diabetes specific interprofessional education (IPE) course and the test scores of matched control students (control) not participating in the course

Area of targeted competence^a^	IPE students						Control			Significance of differences (*p*)			
	Pre‐course (Nurse *n* = 15, Med *n* = 15)			Post‐course (Nurse *n* = 11, Med *n* = 14)			Students (Nurse *n* = 14, Med *n* = 15)			Pre‐post^b^	IPE‐control^c^	Nurse‐med^c^	
	Md	Min	Max	Md	Min	Max	Md	Min	Max			Pre	Post
Diabetes prevention												.272	.106
Nurse	3	2	5	4	1	5	4	3	5	.130	.548		
Med	4	3	5	4	2	5	4	3	5	1.000	.020*		
Diabetes diagnosis & epidemiology												.009*	.185
Nurse	3	2	4	3	1	4	4	3	5	.477	.006*		
Med	4	3	5	4	3	5	4	3	5	.739	.202		
Diabetes assessment, care & follow‐up												.002*	.929
Nurse	3	1	4	4	3	5	4	3	5	.020*	.289		
Med	4	3	5	4	3	5	4	3	5	.739	.775		
Medication												.002*	.449
Nurse	3	1	4	4	1	5	5	3	5	.031*	.013*		
Med	4	3	5	4	3	5	4	3	5	.480	.574		
Insulin therapy												.012*	.707
Nurse	3	1	4	4	1	5	4.5	0	5	.045*	.047*		
Med	4	3	5	4	2	5	4	3	5	1.000	.240		
Nutrition												1.000	.278
Nurse	3	2	4	4	1	5	5	4	5	.248	.005*		
Med	3	2	4	3.5	3	4	4	3	5	.180	.499		
Exercise												.387	.340
Nurse	3	2	4	4	2	5	4	3	5	.084	.099		
Med	3	0	4	4	2	4	3	2	5	.257	.779		
Foot care												.455	.133
Nurse	3	2	4	4	1	4	4	3	5	.083	.031*		
Med	3	2	4	3	2	4	3	2	5	.034*	.511		
Psychology												.350	.722
Nurse	3	2	4	4	1	4	4	3	5	.083	.110		
Med	3	1	4	3	2	5	3	2	5	.236	.663		
Management of care & rehabilitation												.809	.792
Nurse	3	1	4	3	1	4	4	3	5	.070	.078		
Med	3	1	4	3	0	5	4	3	5	.256	.334		
Individual patient guidance												.473	.028*
Nurse	3	2	5	4	3	5	4	2	5	.014*	.351		
Med	3	2	5	3	2	5	4	1	5	.096	.179		
Patient group guidance												.303	.670
Nurse	2	1	4	3	0	4	4	3	5	.463	.017*		
Med	2	0	3	3	0	4	3	0	4	.103	.802		
Social welfare & legislation												.264	.929
Nurse	3	2	4	3	2	4	4	3	5	.516	.037*		
Med	3	2	4	3	2	4	3	2	5	.013*	.343		

Abbreviations: Md, median; Max, maximum; Min, minimum.

* Statistically significant difference, *p* < .05.

^a^ 0 = no competence, 1 = basic competence, 2 = fair competence, 3 = proficient competence, 4 = highly advanced competence, 5 = specialized competence.

^b^ Wilcoxon Signed Rank test.

^c^ Mann–Whitney *U* test.

### 4.4 Students' perceptions of their desired competence in interprofessional diabetes management

The qualitative findings of the students' perceptions of their desired competence in diabetes management are illustrated as “Health care professional as a continuous learner” with four *subcategories* (Table 3). The subcategory *Assuring the theoretical competence* reflects the students' intent to maintain the acquired knowledge and to deepen it further. Many students were concerned about how easily the knowledge may disappear. They wanted to gain more comprehensive knowledge and understanding of individualized diabetes management, i.e., how to operate in diverse situations with diverse patients.

**TABLE 3:** Nursing and medical students' perceptions of achieving their targeted competence in diabetes management

Code	Subcategory	Category
Maintaining information Cannot remember Swotting up information Forgetting things Need for reviewing the information Lack of repeating Need for additional knowledge More comprehensive learning Need of knowledge of diverse situations More of individualized care	Assuring the theoretical competence	Healthcare professional as a continuous learner
Practice in real life Practicing the learned Attempt, error and learning Learning in working life Working life teaches the rest Repetition of patient cases Doing things for real Learning from real life Competency comes from real life Practicing brings certainty	Clinical competence	
Tight criteria of diabetes competence An extensive amount of information Diabetes is a big entity Setting lower target levels of competence Level 3 as proficient professional Understanding if targets are not achieved Rescuing all cannot be a target Continue to learn more	Awareness of one's current competence	
Able to focus on a variety of things Supporting diabetes prevention Weight‐management counselling Supporting self‐care Motivating in lifestyle issues Encouraging in self‐care Concern of the patient Avoiding worsening of the disease Interference in time Sad patient stories	Competency as help for patients

There's so much of this information, and of that knowledge; you should have a comprehensive competence here. (focus group 2)

Therefore, the *Clinical Competence* and practice were considered crucial. Applying the learned abilities to clinical practice, through various patient cases was considered the ultimate and only real learning context.

So that you are able to really do these things and attempt and try and err and try again, that's where the real learning comes from. (focus group 3)

*Awareness of One's Current Competence* reveals the students' notion that the criteria for specialized competence were remarkably high, setting the average level of 3 to represent a really good professional level. Therefore, some students set their targeted competence level after the course lower than before the course. They noted that diabetes management contains an extensive amount of information, but were not discouraged, if they could not achieve all their targets during the course. Instead, they were looking forward to learning more in the future.

Quite many targets remained unachieved, but it wasn't too discouraging. (focus group 3)

Finally, the subcategory *Competency as Help for Patients* contains students' expressions of concern about the potential negative burden of diabetes in the patients' life and how they can and will use their competence to support and motivate the patients in their self‐management and to prevent the complications of diabetes.

That's a fate you don't hope for anyone (a complicated patient case) … It's then easier to begin to encourage and motivate the patient to make these changes in the lifestyle. (focus group 1)

## 5 DISCUSSION

This study evaluated the impact of a novel diabetes‐specific IPE course on undergraduate healthcare students' diabetes knowledge, self‐evaluated competence and insight into their desired future competence in diabetes management. The IPE intervention improved the students' self‐evaluated competence and the nursing students' in‐depth diabetes knowledge. The baseline gap between the nursing and medical students' self‐evaluated competence in diabetes care disappeared. The participating students showed realistic understanding of their current competence level, self‐regulation competency, motivation to learn more and intent to use the learned abilities in clinical diabetes care.

We followed the principles of mixed methods research and integration through merging; comparing, evaluating and discussing the qualitative and quantitative findings together and against each other (Fetters et al., 2013) and in relation to previous literature. The quantitative and qualitative findings of this study were congruent in many parts and merging of them offered further explanation, as well as complementary aspects to the results.

The IPE course improved the nursing and medical students' self‐evaluated competence to deliver interprofessional diabetes care and the nursing students' in‐depth knowledge of diabetes, indicating deeper understanding of their own discipline‐specific role, as well as the roles of other disciplines in diabetes management. These findings are in line with previously reported outcomes of IPE (e.g., Kangas et al., 2018; Račić et al., 2017; Reeves et al., 2017) and may support positive attitudes towards other healthcare professions and collaborating with them (Fox et al., 2018). The students still desired to learn more, indicating that the course increased the students' interest in interprofessional diabetes care, which was also congruent with the qualitative findings. This interest in learning more from and about other professions is the foundation of IPE, aiming to develop collaborative competencies (Interprofessional Education Collaborative, 2016; World Health Organization, 2010). IPE has been supported to be integrated with interprofessional experiences in authentic clinical practice settings (Kangas et al., 2018; Naumann et al., 2020; Tervaskanto‐Mäentausta et al., 2017). Our findings may stem from the course's practical approach, utilizing the clinical experiences during the course. Various patient cases were encountered in real healthcare settings and were discussed immediately with the professionals present and afterwards in interprofessional seminars with experts from several professions (Kangas et al., 2021). This kind of experiential active learning, with clinic visits and case presentations, has been considered particularly valuable in IPE settings (Darlow et al., 2016).

The non‐participating control nursing students evaluated their current competence higher than the course participants, although they scored lower in the diabetes knowledge test. After the course, the participants seemed to have a better understanding of their skills and competences in diabetes management, compared with the non‐participating control students. This result is in line with IPE's learning objectives, aiming at students recognizing their limitations in skills, knowledge and abilities (Interprofessional Education Collaborative, 2016). The contradictory findings of the self‐evaluations and the factual knowledge tests underline the importance of objectively testing the knowledge and competence of undergraduate students, to ensure a realistic view of their competences.

The participating students also targeted at lower competence levels when compared to the non‐participating control students. This was more apparent in the nursing than in the medical students. The qualitative findings offered more insight into this issue. The course participants found interprofessional diabetes care challenging and complex. They also acknowledged their own incomplete competence and recognized the skills and knowledge still to be learned, especially in real life. Recently, IPE implemented in realistic clinical settings has been shown to enhance students' respect for interprofessional clinical practice (Holmes et al., 2020), which was evident in our course participants, as well. Recognizing one's own competence in relation to other team members is essential for effective interprofessional team functioning, when these students work as professionals in the healthcare system (Center, 2018).

The results indicate a remarkable potential of nursing students to adopt in‐depth diabetes knowledge and interprofessional competence in this kind of educational initiatives. The IPE course significantly balanced the baseline difference between the nursing and medical students' self‐evaluated competence in diabetes care. In line with our results, a previous study found IPE effective especially in the education of nursing students, indicating its potential in preparing collaborative‐ready graduating nurses (Thompson et al., 2020). Our findings revealed the students' positive attitude towards learning more and showed how highly the students value clinical competence and learning in practice. The students also expressed an intent to use their acquired competencies to help the patients. For the nursing students this patient‐centred approach was especially congruent with the quantitative results showing significantly higher targeted competence levels regarding Individual Patient Guidance, which was the only post‐course difference in targeted competence compared with the medical students.

Interestingly, the students' perceptions of their desired competence in diabetes management in the future, e.g., *Competency as help for patients* and *Assuring theoretical competence*, related closely to the qualities expected from healthcare professionals, i.e., the core principles of professionalism (Byram, 2017, p. 9). For physicians, these principles include the promise to serve the patient, to acquire, maintain and advance knowledge and skills, life‐long learning and professional formation (American Board of Medical Specialties, 2012). Similarly, the Ethical Guidelines of Nursing also promote constant evaluation and development of one's own competence and intent to improve the patients' quality of life (Finnish Nurses Association, 2021). These findings illustrate the level of the professional identity of nearly graduated students and their adoption of the professional core standards in health care. Even though these findings cannot be considered a result of a single interprofessional course, it is delightful to notice the professional growth related to the intentions to develop further and to use the acquired knowledge and skills in clinical practice

### 5.1 Strengths and limitations

The strengths of this study are in the interprofessional course and the study protocol, which were carefully planned and successfully organized by an interprofessional group specialized in diabetes care, education and research. As previous studies have mainly focused on healthcare professionals, this study significantly enhanced our knowledge regarding the diabetes competence of undergraduate students. The mixed‐methods design can also be seen as a strength of this study, as combining qualitative aspects with the quantitative results offered valuable insight into the subject. A coherence of the results was observed, i.e., different forms of data resulted in similar findings, increasing their credibility.

The study also has some limitations. This study is based on a relatively small number of students, due to the voluntary pilot course setting, and the labour‐intensive learning methods applied. As previously stated, planning and conducting an IPE programme generally requires extensive efforts and resources (Kangas et al., 2018; Lawlis et al., 2014). In addition, the course participants were selected by their own willingness and did not necessarily represent the whole undergraduate student population, which may diminish the generalizability of the results. The applied diabetes knowledge test and self‐assessment tool are not officially validated. The self‐evaluation tool, however, has been designed by the Finnish Diabetes Association and used in clinical practice to evaluate the diabetes competence of healthcare professionals (Unpublished results).

## 6 CONCLUSION

This IPE intervention increased the students' self‐evaluated competence, interest in specific areas of interprofessional diabetes management and nursing students' in‐depth diabetes knowledge, offering a good starting point for interprofessional collaboration in diabetes management. The non‐participating students tended to evaluate their competence higher and to target at higher competence levels than the participants, although the nursing students' scores were lower in the diabetes knowledge test. The findings revealed the participating students' realistic understanding of their current competence level, motivation to learn more and intent to use the acquired knowledge and skills in clinical diabetes care. In addition, it gave an insight into the nearly graduated healthcare students' level of professional identification and self‐regulation competency. The sustainability and generalizability of the observed findings deserve to be clarified in further studies.

## AUTHOR CONTRIBUTIONS

All authors have fully met criteria of authorship. They have made substantial contributions to conception and design, or acquisition of data, or analysis and interpretation of data; been involved in drafting the manuscript or revising it critically for important intellectual content; given final approval of the version to be published; participated sufficiently in the work to take public responsibility for the content; and agreed to be accountable for all aspects of the work in ensuring that questions related to the accuracy or integrity of any part of the work are appropriately investigated and resolved.

## CONFLICT OF INTEREST

No conflict of interest has been declared by the authors.

## Supporting information


Appendix S1

Appendix S2

Appendix S3

## Data Availability

The data that support the findings of this study are available from the corresponding author upon reasonable request.
