# Supplementary material for: The impact of interprofessional education on students' current and desired competence in diabetes care
Source: Nurs Open. 2022 Jul 26;10(1):264–77. doi: 10.1002/nop2.1301 (PMC9748052; doi:10.1002/nop2.1301)
Supplement: Supplementary file 1 — Appendix S1 [file NOP2-10-264-s001.docx]

**PRE-COURSE THE IPE COURSE POST-COURSE** QUANTITATIVE ANALYSIS QUALITATIVE ANALYSIS

**Comparisons of diabetes knowledge test results**

- PRE-POST within Nurse students, within Med students
- between Nurse & Med PRE, between Nurse & Med POST
- between Nurse POST & Nurse-C, between Med POST & Med-C

**Comparisons of self-evaluated competence & targeted competence**

- PRE-POST within Nurse students, within Med students
- between Nurse & Med PRE, between Nurse & Med POST
- between Nurse POST & Nurse-C, between Med POST & Med-C

**POST-**

**evaluation**

**and interviews**

Diabetes knowledge test

Self-evaluation in 13 competence areas of diabetes management

Focus-group interviews of 3 mixed groups

**Interactive seminars and lectures**

Case presentations by students

Short expert presentations

Lectures on subjects requested by students

**Nursing and medical students’ collaborative clinic visits**

Two half a day working visits (gerontological ward and diabetes outpatient clinic)

Clinical work, group discussions

Preparing a case presentation

**Students’ self-studies**

**PRE-evaluation and interviews**

Diabetes knowledge test

Web-based Self-evaluation in 13 competence areas of diabetes management

**Starting seminar**

Introduction to the course content and the associated study

Getting acquainted with each other

**→**

**Focus-group interviews** after the course:

Inductive content analysis of students’ perceptions of their current and future diabetes competence

**CONTROLS**

Matched peer students not participating in the IPE course

Diabetes knowledge test

Self-evaluation in 13 competence areas of diabetes management

CONVERGENT PARALLEL DESIGN OF

MIXED METHODS RESEARCH:

Separate analyses and merging in the discussion when applicable

Abbreviations: Participating nursing (Nurse) and medical (Med) students and non-participating control students (Nurse-C, Med-C), interprofessional education (IPE), before the course (PRE), after the course (POST).
