# Supplementary material for: The impact of interprofessional education on students' current and desired competence in diabetes care
Source: Nurs Open. 2022 Jul 26;10(1):264–77. doi: 10.1002/nop2.1301 (PMC9748052; doi:10.1002/nop2.1301)
Supplement: Supplementary file 2 — Appendix S2 [file NOP2-10-264-s002.docx]

Supplementary material 2: Diabetes Knowledge Test

1. What is the most relevant difference between the two main types of diabetes: Type 1 diabetes and type 2 diabetes? max 2p
2. What kind of a diet is recommended for a patient with diabetes? max 4p
3. What is basal insulin and how many times a day should it be injected? max 2p
4. What is the purpose of rapid-acting or short-acting insulin? max 1p
5. How do you estimate the dose of mealtime (rapid-acting) insulin? max 2p
6. Which blood glucose measurements do you need to evaluate, whether the basal insulin dose is right or not? max 2p
7. Which blood glucose measurements do you need to evaluate, whether the mealtime (rapid-acting) insulin doses are appropriate? max 2p
8. What is the target level of fasting blood glucose? max 1p
9. What is the pre-meal target glucose level? max 1p

1. What is the post-meal target glucose level? max 1p
2. Can you skip your basal insulin in some situations? max 1p
3. How do you define severe hypoglycaemia? max 1p
4. How is severe hypoclycaemia managed at home? How is it treated in the emergency room? max 3p
5. How do you advise a person with type 1 diabetes for sick days, for example what he/she should do when he/she has the flu with a fever? max 3p
6. When should ketones be measured? max 2p
7. How do you modify the diabetes medication, when a person with type 2 diabetes is hospitalized for an acute condition? max 1p
8. What is the usual HbA1C target for persons with diabetes? max 1p
9. What are the reasons for adjusting the individual HbA1C target at a higher level than usual? max 4p
10. What are the most important complications of diabetes? max 3p

1. By which means do we attempt to diagnose the complications of diabetes at an early stage? max 3p

Total 40 points
