# Supplementary material for: The impact of interprofessional education on students' current and desired competence in diabetes care
Source: Nurs Open. 2022 Jul 26;10(1):264–77. doi: 10.1002/nop2.1301 (PMC9748052; doi:10.1002/nop2.1301)
Supplement: Supplementary file 3 — Appendix S3 [file NOP2-10-264-s003.docx]

| **Meaning unit** | **Condensed meaning unit** | **Code** | **Sub-category** | **Category** |
| --- | --- | --- | --- | --- |
| ”that you maintain the information you have gained here”  ”I still cannot remember all about medicational things”  ”now it only calls for swotting up these things into your head”  ”surely many of these things will fall out of my head before the summer… ”  “… so you should rehearse it somewhere”  ”it´s a shame that you don´t hammer it (information) in that much now”  “…you should hammer it (information) in quite a lot so that it would stay in your head” | maintain the gained information  cannot remember medication  you should swot up information  things will get forgotten  you shoud rehearse  shame you don´t hammer it in  you should repeat it a lot | maintaining information  cannot remember  swotting up information    things get forgotten  need for rehearsing  lack of rehearsing  need to repeat | Maintain the knowledge | HEALTHCARE PROFESSIONAL AS A CONTINUOUS LEARNER |
